# Top-Down Beta Rhythms Support Selective Attention via Interlaminar Interaction: A Model

**DOI:** 10.1371/journal.pcbi.1003164

**Published:** 2013-08-08

**Authors:** Jung H. Lee, Miles A. Whittington, Nancy J. Kopell

**Affiliations:** 1 Department of Mathematics & Statistics, Boston University, Boston, Massachusetts, United States of America; 2 Department of Neuroscience, Hull York Medical School, Heslington, York, United Kingdom; University of Nottingham, United Kingdom

## Abstract

Cortical rhythms have been thought to play crucial roles in our cognitive abilities. Rhythmic activity in the beta frequency band, around 20 Hz, has been reported in recent studies that focused on neural correlates of attention, indicating that top-down beta rhythms, generated in higher cognitive areas and delivered to earlier sensory areas, can support attentional gain modulation. To elucidate functional roles of beta rhythms and underlying mechanisms, we built a computational model of sensory cortical areas. Our simulation results show that top-down beta rhythms can activate ascending synaptic projections from L5 to L4 and L2/3, responsible for biased competition in superficial layers. In the simulation, slow-inhibitory interneurons are shown to resonate to the 20 Hz input and modulate the activity in superficial layers in an attention-related manner. The predicted critical roles of these cells in attentional gain provide a potential mechanism by which cholinergic drive can support selective attention.

## Author Summary

Top-down signals originate from higher cognitive areas such as parietal and prefrontal cortex and propagate to earlier stages of the brain. They have been thought to be associated with selective attention, and recent physiological studies suggest that top-down signals in the beta frequency band can support selective attention. In this study, we employ a computational model to investigate potential mechanisms by which top-down beta rhythms can influence neural responses induced by presentation of stimuli. The model includes several cell types, reportedly crucial for generating cortical rhythmic activity in the gamma and beta frequency bands, and the simulation results show that top-down beta rhythms are capable of reproducing experimentally observed attentional effects on neural responses to visual stimuli. These modulatory effects of top-down beta rhythms are mainly induced via activation of ascending inhibition originating from deep layer slow inhibitory interneurons. Since the excitability of slow interneurons can be increased by cholinergic neuromodulators, these interneurons may mediate the effects of cholinergic tone on attention.

## Introduction

It is widely understood that sensory processing is modulated by attention, which impacts neural responses in the sensory cortex: Elevated spiking activity [1]–[4] and enhanced synchrony in neural responses [5]–[9] were found to be associated with attended, rather than unattended stimuli. These findings suggested that endogenous signals, presumably generated at least in part in higher cognitive areas, are delivered to lower areas when attentional gain control is required. Although neural correlates of attentional gain control are not well understood, biased competition has been thought to be an underlying mechanism [10]–[17].

Recent studies indicate that beta rhythms can be associated with top-down attention [18]–[23]. In this study we used a computational model to address whether top-down beta rhythms can bias competition, and if so how they achieve this. We leave for a following paper the potential roles of top-down signals in the gamma frequency band, which have also been seen [24], [25], considering here only the induction of gamma rhythms by bottom up signals and how they interact with the top-down beta. Beta rhythms have been reported to be generated by local circuits in deep layers, particularly layer 5 (L5) [24], [26]–[28]. A recent *in vitro* study found that three types of deep layer cells (intrinsically bursting (IB), regular spiking (RS) pyramidal cells and a particular class of slow-inhibitory interneuron (LTS cells)) are involved in generating deep layer beta rhythms locally in the primary auditory cortex [24], and that beta rhythms generated in higher order (parietal) cortices influence rhythm generation in auditory cortex in a highly direction-specific manner.

Cortical slow-inhibitory (SI) interneurons are a diverse subclass of inhibitory cells. Their firing patterns can be regular, accommodating or low-threshold spiking, and their axonal and dendritic morphology also varies greatly from cell to cell. However, the majority of this broad class of interneuron is involved in providing inhibition between cortical layers that has slow postsynaptic kinetics relative to fast spiking interneurons. For example deep layer Martinotti cells have axons that are almost exclusively oriented radially in cortex, passing across multiple local laminae [29], [30]. In addition, Dantzker & Callaway found a class of adapting interneurons in superficial layers that received dominant inputs from deep layers [31]. These factors make SI interneurons ideal candidates for mediating interlaminar interactions, as has been shown for concatenation of deep and superficial beta and gamma rhythms [32]. Additionally, the excitability and spike output patterns in SI interneurons can be potently affected by cholinergic neuromodulation, a cortical process of fundamental importance to attention (see Reference [33] for review). Specifically, Xiang et al. [29] found that acetylcholine depolarized deep layer LTS interneurons, which can enhance interlaminar interaction. Thus, we hypothesized that primary sensory L5 cells, resonating to top-down beta frequency inputs can modulate responses of superficial neurons in sensory cortices predominantly through SI interneurons. The model given below supports this hypothesis.

## Results

Fries et al. [5], [6] proposed an experimental scheme capable of observing modulation of neural activity induced by top-down attention. They trained monkeys to pay attention to one of two stimuli presented simultaneously, while monkeys maintained fixation. By comparing neural activity when monkeys paid attention to a stimulus inside the receptive field to when monkeys paid attention to a stimulus outside the receptive field, they found that top-down attention enhanced firing rate and modulated local field potentials (LFPs). More specifically, attention enhanced spike-field coherence in the gamma frequency band (30–70 Hz) but reduced it in frequencies lower than 17 Hz [5], [6]. To simulate two different attentional conditions-“attention-inside the receptive field (RF)” and “attention-outside the RF”- we built two cortical columns, each corresponding to one of these two attentional conditions. One column receives both top-down and bottom-up signals, and another receives bottom-up inputs only; thus the two columns are associated with the attended stimulus and unattended one, respectively. For brevity, we shall refer to these as the “attended column and the unattended column”. In addition, L2/3 RS cells of both columns receive background inputs. In this study, top-down signals are synchronous synaptic inputs, whereas bottom-up and background inputs are asynchronous synaptic inputs (see Methods).

In the hierarchical structure proposed by Felleman & Van Essen [34], [35], top-down signals target superficial and deep layers while they avoid granular layers. Other studies suggested that top-down signals mainly project to superficial layers [23], [36]. In either circumstance deep layer pyramidal cells can access top-down signals: Even if top-down signals project to superficial layers only, they can innervate L5 pyramidal cells via vertical apical dendrites [37]. Thus, we introduced top-down signals in the beta frequency band into L5 pyramidal cells. Importantly, Roopun et al. [24] suggested that superficial and deep layers may receive distinctive top-down signals: superficial layers receive top-down gamma rhythms, whereas deep layers receive top-down beta rhythms, consistent with other studies [28], [35]. In this study, we focused on the effect of top-down beta rhythms on neural responses by introducing top-down beta rhythms into L5 pyramidal cells of the attended column; we leave to a forthcoming paper, describing effects of top-down gamma rhythms, a description of the effects of top-down signals to the superficial layers (see Discussion).

**Figure 1:**
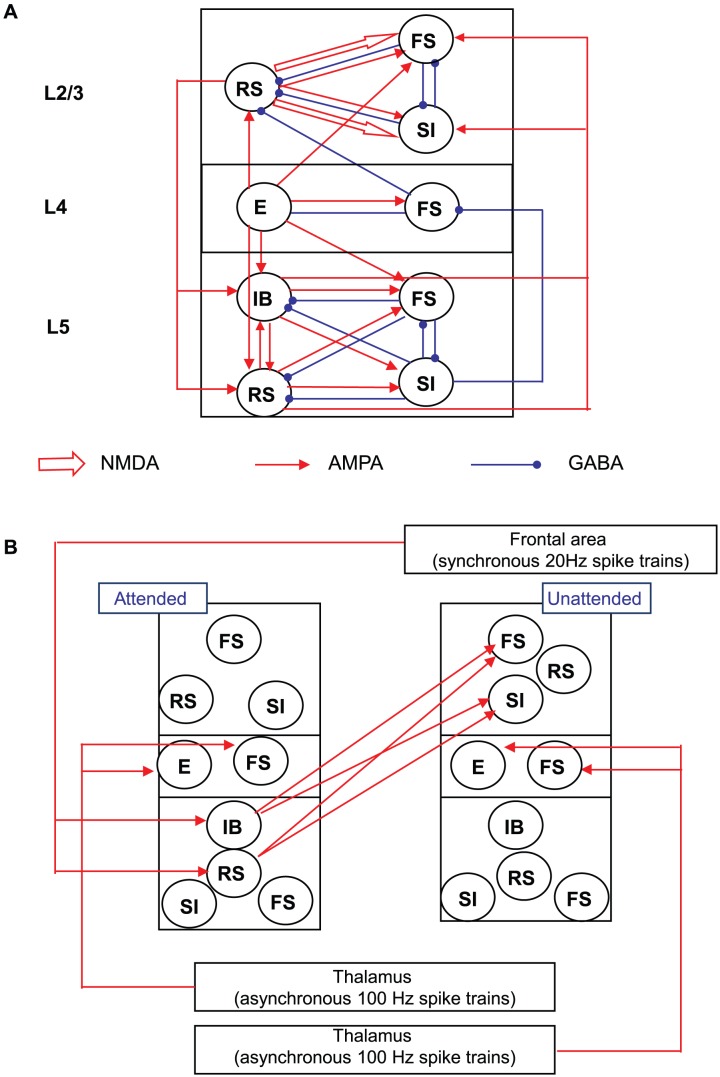
The structure of the model. (A). Structure of a single column. Each circle is a population of 20 cells. Open and solid arrows represent NMDA and AMPA synapses respectively. Blue circles are GABA synapses. (B). Full model. Two columns interact with each other via ascending excitatory synapses from L5 pyramidal cells to L2/3 FS and SI cells. Both columns receive 100 Hz Poisson EPSC trains. Synchronous top-down signals are introduced into the attended column only. For clarity, we do not display recurrent connections inside the population of the same type: all cells interact with others that belong to the same population (see Methods).

We modeled cortical columns with superficial (layer 2/3), granular (layer 4) and deep (layer 5) layers (see Figure 1), providing a reduced model of a biophysically detailed model proposed in earlier computational works [24], [38]. For brevity, we will refer to layer A as “LA” where A is the label. Figure 1 shows the connectivity among 9 cell populations of each type. We minimized bias from random connectivity and random noisy inputs by using 10 simulations, each of which used a different realization of our model and noisy inputs. The superficial layer, representing L2 and L3, consists of regular spiking pyramidal cells (RS), fast spiking interneurons (FS) and slow inhibitory interneurons (SI). L4 contains E (excitatory) cells, which models both stellate and pyramidal cells in this layer, and FS interneurons. For L5, we implemented two types of pyramidal cells, intrinsic bursting (IB) and regular spiking (RS), and SI interneurons, cell types that were active in cholinergically induced *in vitro* beta rhythms in the primary auditory cortex [24]. In addition, the deep layer also contains FS interneurons. Each cell of our model receives both excitatory and inhibitory synaptic inputs from various cell types via both intralaminar and interlaminar connections (see Methods and Figure 1). We connected the two columns with excitatory synapses from L5 pyramids to L2/3 interneurons. These intercolumnar connections are identical to intracolumnar connections from L5 pyramids to L2/3 interneurons except that intercolumnar connections to L2/3 SI cells are stronger than intracolumnar connections by 50%. Details about the connectivity are in Methods. In our model, L5 pyramidal cells excite L2/3 interneurons, and L5 SI cells inhibit L4 FS cells (see Figure 1). The former and latter will be referred to as “ascending excitation” and “ascending inhibition”.

Our simulation paradigm follows Fries et al. [5], [6], who presented a cue followed by the delay and stimulus periods, and found that top-down attention modulated neural responses in both periods. Thus, we simulated the delay and stimulus periods, respectively. The data of Fries et al. [6] was presumed by the authors to come mainly from superficial layers, as was the in vivo data from Wang [39]. Thus, we evaluated the effect of top-down beta rhythms on L2/3 activity from 10 independent simulations. In particular, we calculated the spike-triggered average (STA) of L2/3 local field potentials (LFPs simulated by summing up synaptic inputs to pyramidal cells, see Methods) and RS cell spiking activity, and compared these neural responses between the attended and unattended columns. To do so, we used attentional indices (see Methods), suggested in Fries et al. [5]: Two attentional indices, AI(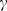) and AI(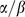), measure difference in spike-field coherence (SFC) between attentional conditions, and the third attentional index, AI(R), estimates difference in the firing rate (R) (see Methods). As in Fries et al. [5], attentional indices are positive (see Methods) when a stronger response is observed in the attended column. Since SFC estimates the synchrony of neural responses [5], AI(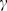) and AI(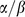) allow us to evaluate the effect of top-down beta rhythms on the synchrony in neural responses. In this study, AI(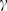) and AI(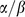) measures synchrony in the gamma (25–70 Hz) and the alpha/beta (8–25 Hz) frequency bands. Additionally, we ran simulations without specific components of our model so that we can clarify functional roles of those components of our model.

### Delay period: Activity with top-down, but without bottom-up, input

During the delay period, Fries et al. [5], [6] did not present a stimulus, but they found disparate neural responses in the attention-inside the RF condition and in the attention-outside the RF condition: LFP power in frequencies lower than 17 Hz was smaller in the attention-inside RF trials than in the attention-outside RF trials. Such differences in neural activities were attributed to top-down attention, which was induced by a cue preceding the delay period. We therefore tested whether top-down beta rhythms could reproduce these neurophysiological findings.

#### Control experiment: Activity induced background inputs only

**Figure 2:**
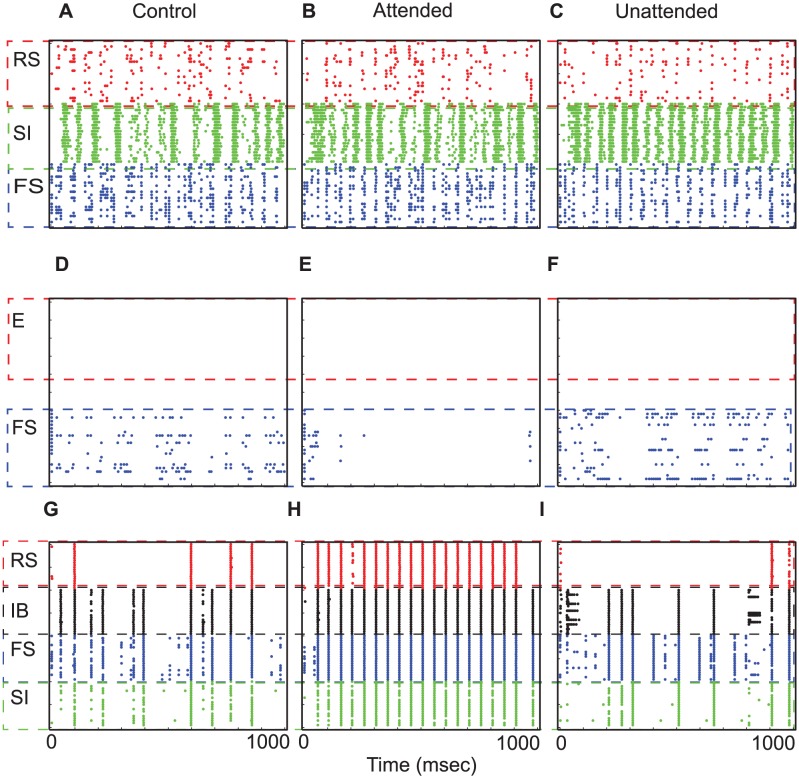
Cell activity in response to top-down beta rhythms. (A)–(I). Neural responses of the control (without bottom-up or top-down inputs), attended and the unattended columns. Each dot represents an action potential. x-axis shows simulation time, and y-axis displays the cell number.

As a control experiment, we first examined the neural activity without introducing top-down or bottom-up inputs. Thus, the two columns were both driven by background inputs. The first column of Figure 2 shows neural responses of our model in this control experiment, without top-down or bottom-up inputs. Background inputs (see Methods) induce L2/3 RS cells to fire sporadically, which in turn drives L5 IB cells to fire a few times via descending excitation. L5 cells fire synchronously due to slowly decaying inhibition of L5 SI cells and recurrent synaptic connections among L5 IB cells. As can be seen in Figure 2G, IB cells fire for a few cycles of 10 Hz alpha rhythms.

#### L5 cells of the attended column resonate to top-down beta rhythms

**Figure 3:**
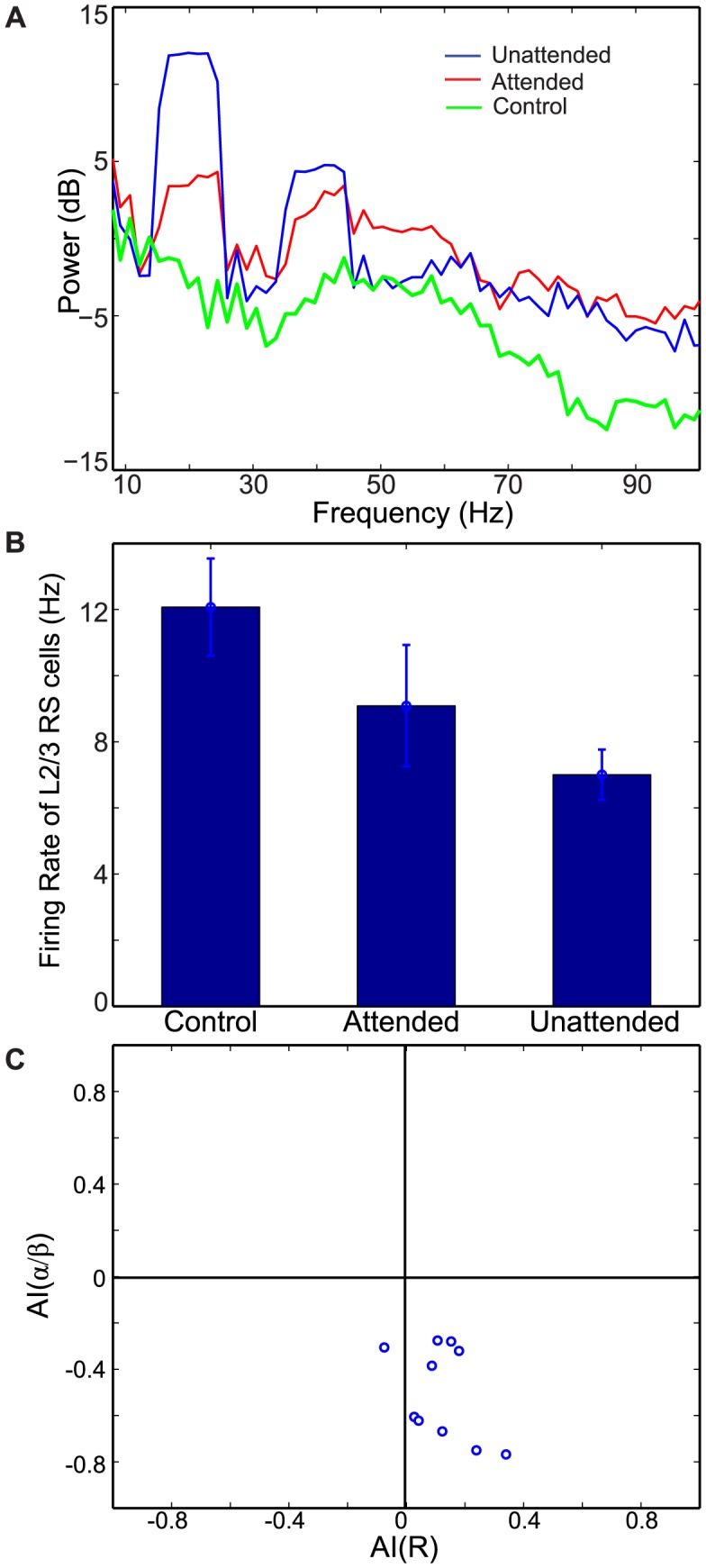
The effect of top-down beta rhythms on neural responses. (A). The power spectra of STA of LFPs in all three conditions. (B). The average firing rate of L2/3 RS cells from 10 simulations, and the error bar is the standard deviation of 10 simulations. (C). Scatter plots of attentional indices; AI(R) on x-axis, AI(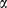/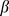) on y-axis.

Top-down signals, 20 Hz synchronous synaptic inputs, are introduced to L5 pyramidal cells of the attended column only, and the attended and unattended columns generate disparate neural responses. The second and third columns of Figure 2 show raster plots of the two columns, respectively, and Figure 3 displays STA of LFPs and L2/3 RS cell spiking activity from 10 simulations. In the attended column, L5 IB and RS cells, which receive top-down signaling, oscillate at 20 Hz (Figure 2H) and project beta rhythmic excitatory synaptic inputs divergently to L2/3 FS and SI cells of both columns, whereas L5 SI cells of the attended column project beta rhythmic inhibitory synaptic inputs to L4 FS only within the column. Because of this top-down signaling, L5 SI cell activity is much stronger in the attended column than in the unattended column, producing disparity in ascending inhibitory projections to L4 FS cells between the two columns. Since, in our model, intercolumnar projections to L2/3 SI cells are stronger than those within the column (see Methods), L2/3 SI cells show stronger spiking activity in the unattended column than in the attended column, and thus stronger inhibition is induced in the unattended column, generating disparity in STA of LFPs (Figure 3A) and spiking activity of L2/3 RS cells (Figure 3B); we note that superficial cells fire mostly at the cycles of beta rhythms, and therefore the second peak in Figure 3A should be considered as a harmonic of 20 Hz rhythm. These results indicate that top-down beta rhythms can suppress L2/3 RS cell activity induced by background inputs.

Spike field coherence (SFC) in the alpha/beta frequency band is also modulated by top-down beta rhythms. As can be seen in Figure 3C, attentional index AI(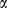/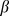), which measures difference in synchrony in frequencies from 8 to 25 Hz between the two columns (see Methods), is significantly smaller than 0 (t-test, 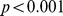), indicating that top-down beta rhythms reduce the synchrony in these low frequencies. These effects of top-down beta rhythms, consistent with attentional effects reported in Fries et al. [5], are mainly attributed to the fact that L2/3 SI cell activity is enhanced more strongly in the unattended column, due to intercolumnar projection from L5 pyramidal cells of the attended column.

#### L2/3 SI cells help suppress non-stimulus evoked activity

**Figure 4:**
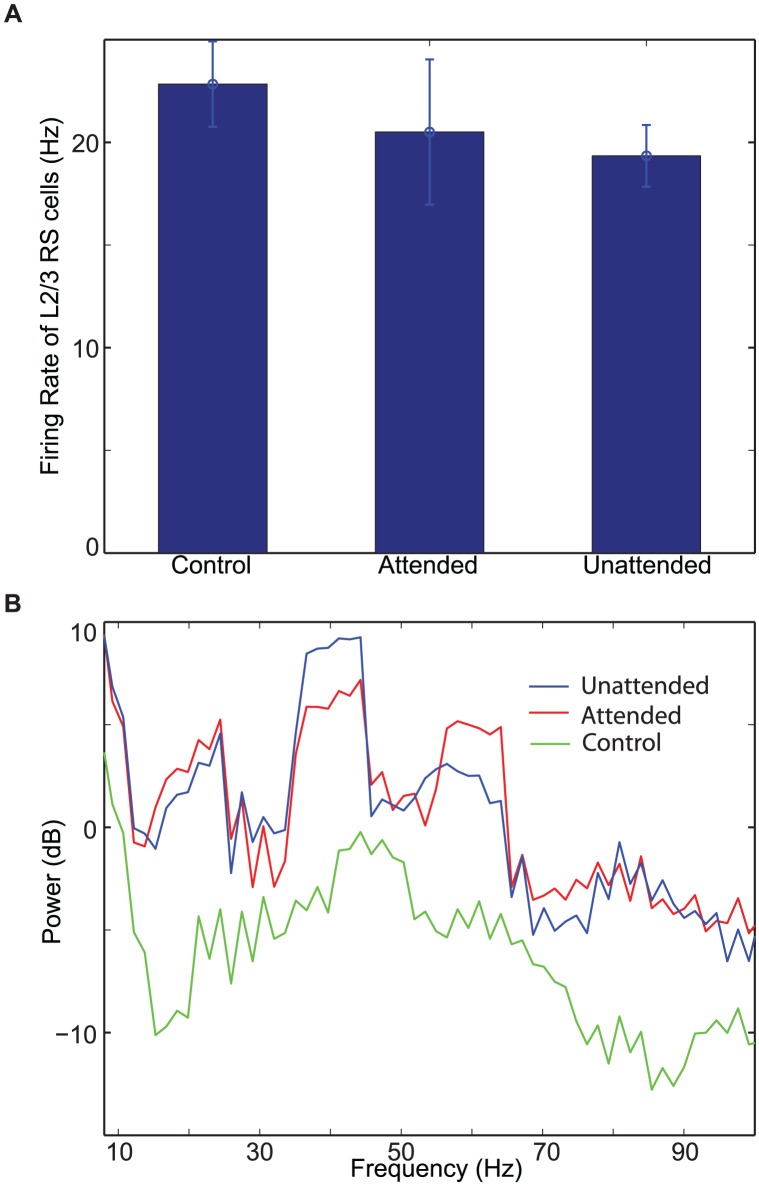
The effect of top-down beta rhythms on neural responses without L2/3 SI cells. All parameters are the same as in Figure 3, but with SI cells removed from the network. (A). The firing rate of L2/3 RS cell activity from 10 simulation. (B). The power spectra of STA of LFPs.

L2/3 SI cells of our model receive strong excitatory projection from deep layer cells of both columns, suggesting that L2/3 SI cells are critical for generating superficial beta rhythms. To investigate the functional role of superficial beta rhythms, we removed L2/3 SI cells from both columns. L2/3 RS cell activity is enhanced in all conditions (control, attended and unattended), as shown in Figure 4A. Importantly, the difference in firing rates of L2/3 RS cells among attentional conditions becomes smaller, suggesting the pivotal role of L2/3 SI cells in suppression of non-stimulus evoked activity. The STA of LFPs is also changed. Without L2/3 SI cells, L2/3 RS cells produce strong spiking activity and induce gamma-frequency power in all conditions (Figure 4B); without L2/3 SI cells, RS cells fire at a gamma frequency frequently (data not shown), and thus the 40-Hz peak is real rather than a harmonic of 20 Hz as shown in Figure 3A. These results indicate that, in our model, FS cells alone are unable to prevent RS cells from responding to background inputs and thus generating gamma rhythmic activity. In contrast, SI cells, which produce slowly decaying inhibition (see Methods), are suitable for suppressing L2/3 RS cell spiking activity and resulting gamma rhythms provoked by background inputs.

### Stimulus period: Combination of top-down and bottom-up inputs

**Figure 5:**
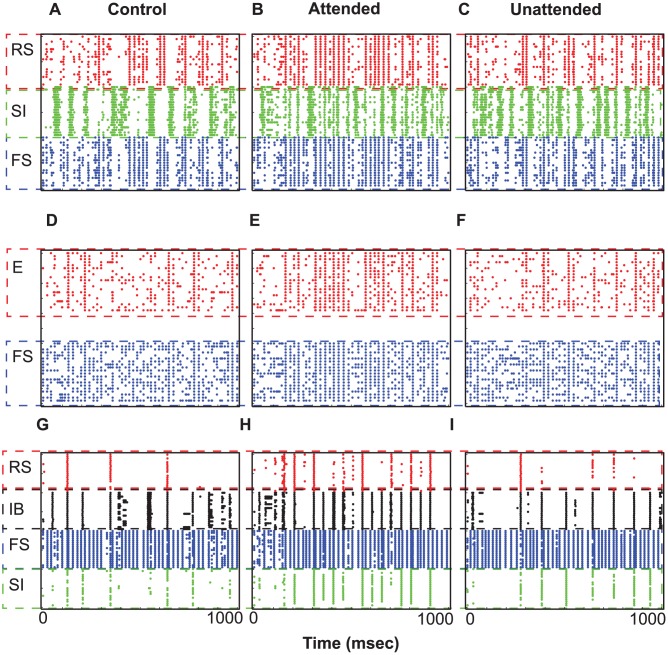
Neural activity during the stimulus period. (A)–(I). Neural responses of the control, attended and the attended columns, respectively.

Fries et al. [5], [6] showed that stimulus presentation generated gamma rhythms and elevated spiking activity, and attention enhanced these induced neural responses. In our model bottom-up inputs, simulated by 100 Hz asynchronous trains of EPSCs, are introduced to L4 E and FS cells of both columns. Without top-down signals, the two columns generate equivalent responses (first column of Figure 5): Even though bottom-up inputs are asynchronous, L4 E cells fire synchronously at a gamma frequency for a few gamma cycles due to interactions between L4 E and FS cells (see References [40], [41] for details). L2/3 also oscillates at the same frequency due to the ascending excitation from L4 E cells to L2/3 RS and FS cells (Figure 5A).

#### Top-down beta rhythms induce disparate neural responses between the two columns

To study the effect of top-down beta rhythms on neural responses induced by bottom-up inputs, we introduced beta rhythms into L5 pyramidal cells of the attended column only while presenting bottom-up inputs to both columns. Although both columns receive equivalent bottom-up inputs, different neural responses are elicited between them in all layers in a manner dependent upon the top-down beta rhythms. The second and third columns of Figure 5 show neural responses of the attended and unattended columns, respectively. As simulations of the delay period show above, top-down beta rhythms induce stronger L2/3 SI cell activity in the unattended column. The mean value and the standard deviation of firing rate of L2/3 SI cells of the unattended column are 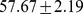 Hz, whereas the mean firing rate are significantly reduced (t-test, 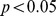) to 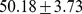 Hz in the attended column. Thus, stronger inhibition is generated in L2/3 of the unattended column (Figure 5B and C). We also note that, in L4 and L2/3, excitatory cells (RS and E) are capable of firing synchronously for a few of cycles of gamma rhythms. Interestingly, they often skip several cycles, generating much slower rhythms close to alpha frequency. As can be seen in Figure 5, L2/3 RS and L4 E cells of the unattended column produced these slower rhythms more strongly.

**Figure 6:**
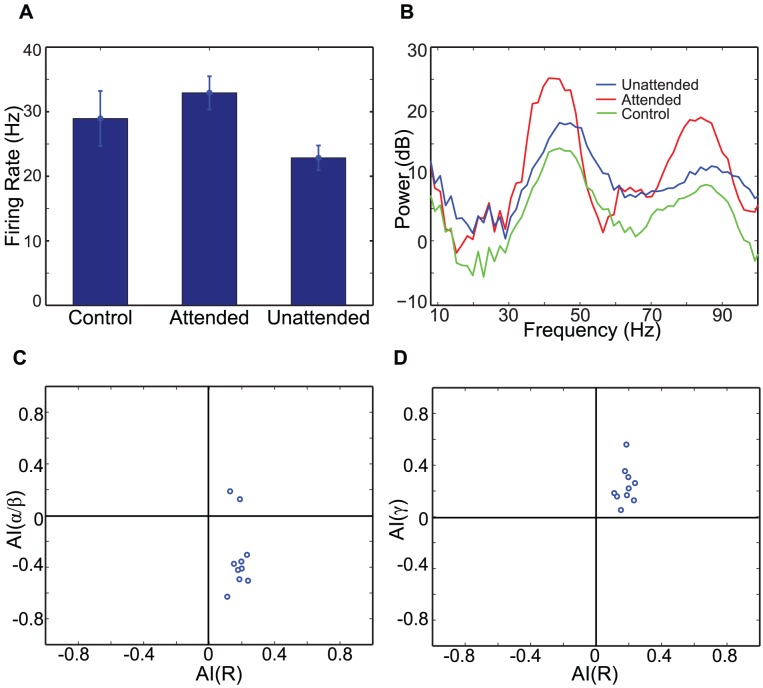
The effect of top-down beta rhythms on neural responses during the stimulus period. (A). The firing rate of L2/3 RS cells. (B). The power spectra of STA of LFPs. A logarithmic scale is used on y-axis. (C)–(D). Scatter plots of attentional indices.

To estimate the effect of top-down beta rhythms without any bias from connectivity and external noise, we ran 10 simulations with different sets of synaptic connections and background inputs. In all 10 simulations, top-down beta rhythms enhance L2/3 RS cell spiking activity of the attended column relative to that of control experiment (Figure 6A), and the t-test confirms that this enhancement is significant (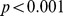). In contrast, the firing rate of L2/3 RS cells is always lowest in the unattended column (Figure 6A). A similar pattern emerges in STA of LFPs: Gamma-frequency power is the highest in the attended column, but low-frequency power is the highest in the unattended column, as shown Figure 6B. We note that the power spectral density of STA of LFPs of the control experiment is lower than others in all frequency bands. These modulatory effects of top-down beta rhythms are consistent with attentional modulation of neural responses, reported in Fries et al. [5]. We further examined the effect of top-down beta rhythms by calculating attentional indices (see Methods) [5]. All three indices (AI(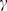), AI(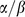) and AI(R)), as shown in Figure 6, are significantly modulated by top-down beta rhythms (t-test, 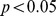), and those patterns of modulation are also consistent with data reported in Fries et al. [5].

#### Ascending inhibition from L5 SI cells enhance gamma rhythms, helping pyramidal cells in L4 and L2/3 to respond reliably to bottom-up inputs

**Figure 7:**
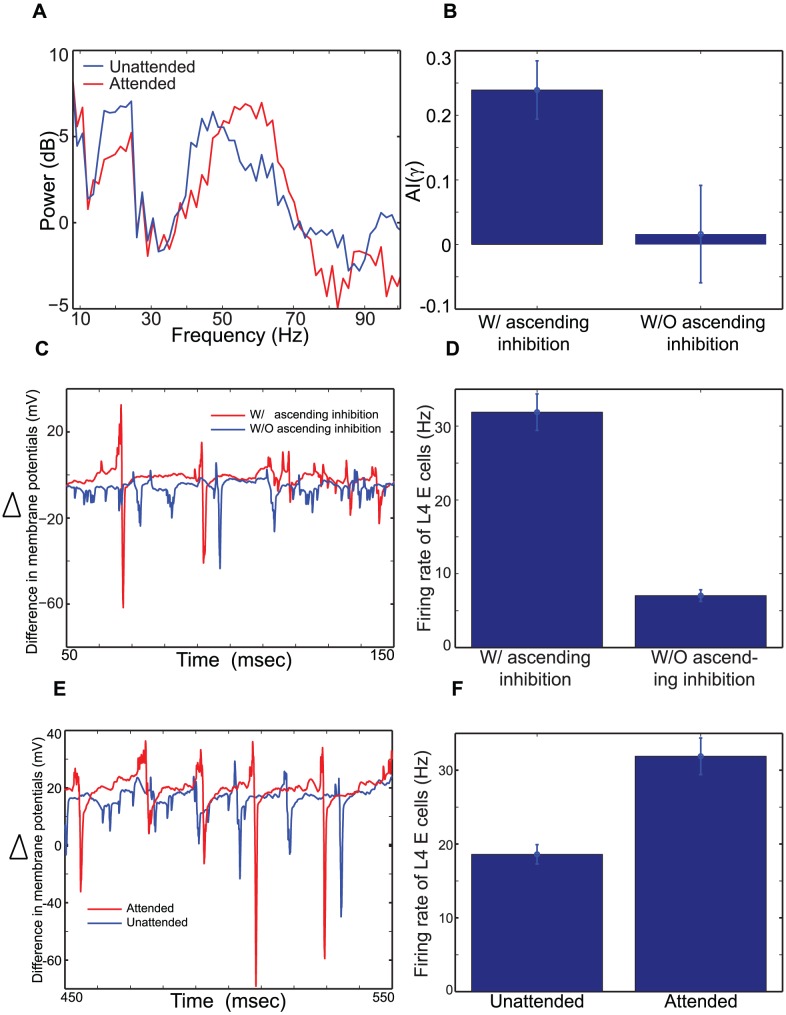
Functional roles of ascending inhibition. (A). The power spectra of STA of LFPs in the attended and unattended columns, without ascending inhibition from L5 SI cells to L4 FS cells. (B). Comparison of AI(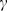), with and without ascending inhibition. (C). Difference in average membrane potentials between L4 E and L4 FS cells, 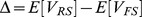, where 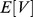 is the mean value of membrane potentials over 20 cells. Thus, positive peaks represent moments when more L4 E cells spike than L4 FS cells, and negative peaks show moments when more L4 FS cells spike more than L4 E cells. The membrane potential difference in the attended column is compared, with and without ascending inhibition. (D). Comparison of the firing rate of L4 E cells of the attended column, with and without ascending inhibition. (E). Comparison of 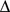 between the attended and unattended columns with ascending inhibition. (F). Comparison of the firing rate of L4 E cells between the attended and unattended columns.

Top-down beta rhythms, which entrain L5 cells of the attended column, can modulate upper neural responses via ascending excitation and inhibition. Since simulations of the delay period above suggested ascending excitation can generate different amounts of inhibition of L2/3 SI cells in superficial layers of the two columns, we first examined functional roles of ascending inhibition by running simulations without the latter. The removal of ascending inhibition reduces the gamma power in STA of LFPs (Figure 7A), and attentional effects on gamma-frequency SFC are reduced; they are not significantly higher than 0 without ascending inhibition (t-test, 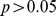), as shown in AI(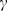) (Figure 7B). These results suggest that ascending inhibition may be critical for attentional effects on gamma rhythms in L2/3.

The importance of ascending inhibition can be understood as follows: A previous computational model showed that asynchronous spiking activity of interneurons can more effectively reduce pyramidal cell activity than synchronous spiking activity [41], suggesting that gamma rhythms can provide widows of opportunity for L4 E cells to fire. In our model, bottom-up inputs generate gamma rhythms (Figure 5E and F) via interaction between pyramidal cells and FS cells. In this mechanism, known as the PING [40], [41], interneuron activity is induced by excitation from pyramidal cells to produce prominent gamma rhythms. However, if FS cells fire independently of pyramidal cell activity, gamma rhythmic activity is disrupted and thus pyramidal cell activity is suppressed [41]. In our model the ascending inhibition regulates the L4 FS cell activity and thus gamma rhythms in L4: When we removed ascending inhibition, L4 FS cells were released from inhibition from L5 SI cells, and thus fire between cycles of gamma rhythms (Figure 7C). The positive peaks in Figure 7C correspond to moments when most E cells fire but not FS cells, and the negative peaks vice versa. When prominent gamma rhythms exist, peaks of difference in membrane potentials come immediately before troughs. With ascending inhibition, prominent peaks come immediately before troughs at most cycles, whereas without ascending inhibition troughs come earlier than peaks much more often. As expected, L4 E cell activity is reduced significantly when ascending inhibition is removed (Figure 7D, t-test, 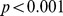).

Importantly, top-down beta rhythms can generate a prominent ascending inhibition only within the attended column. As a result, L4 FS cell activity in the attended column is activated differently from that of the unattended column: L4 FS cell activity of the attended column is induced mostly by L4 E cell activity (Figure 7E), whereas L4 FS cells of the unattended column can spike often without excitation from L4 E cells. Thus, L4 E cell activity is stronger in the attended column than in the unattended column (Figure 7F), allowing L2/3 cells of the attended column to receive stronger (stimulus-dependent) bottom-up inputs from L4 and generate stronger gamma rhythms. These simulation results suggest that top-down beta rhythms can selectively increase L4 excitatory projection to L2/3 cells in response to bottom-up inputs.

#### Connections across columns convey lateral inhibition critical for reduction of synchrony in the low frequency band

As discussed above, ascending excitation, from L5 to L2/3 interneurons, generate stronger inhibition to L2/3 RS cells of the unattended column. In other words, ascending excitation provides lateral inhibition, which is believed to play a critical role in biased competition, a leading candidate for neural correlates of selective attention [3], [10], [13], [42], [43]. In this study, we did not consider lateral inhibition conveyed via superficial to superficial connections (see Reference [44]), since this type of inhibition creates involuntary competition between neural responses induced by bottom-up inputs, which is different from attentional gain control discussed in this study. By contrast, in our model, lateral inhibition is conveyed via ascending intercolumnar excitatory projections controlled by top-down beta rhythms.

**Figure 8:**
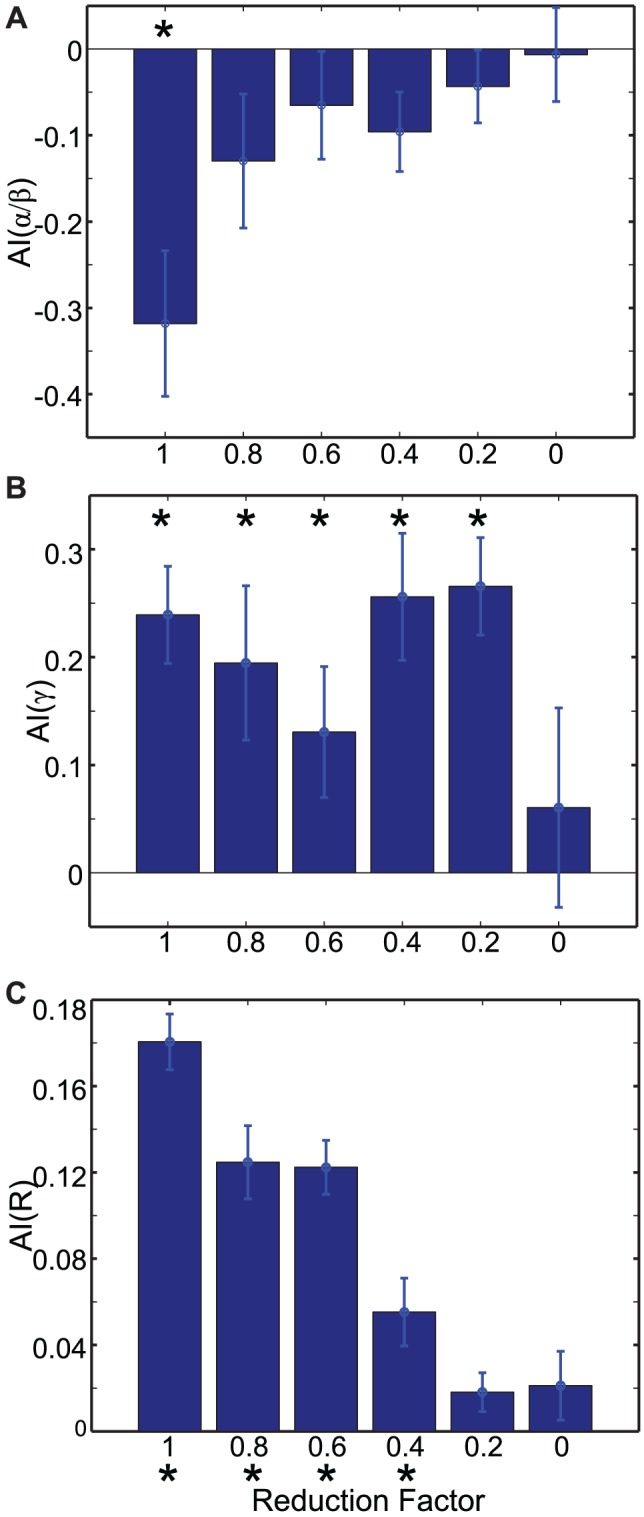
The impact of intercolumnar connections on attentional modulation. Attentional indices with gradually reduced intercolumbar projection. AI(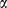/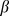), AI(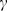), AI(R) are displayed in (A),(B) and (C), respectively. * represents distributions significantly different from 0.

To understand how lateral inhibition affects our results, we gradually reduced the strength of intercolumnar projection and measured attentional indices. Figure 8 displays three attentional indices evaluated with reduced lateral connection. Attentional modulation in the alpha/beta frequency band is most sensitive. When we reduced the synaptic strength of lateral connections by 20%, reduction in AI(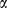/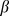) became insignificant (Figure 8A). By contrast, AI(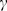) was significantly enhanced (t-test, 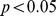) until 20% reduction was made (Figure 8B) and AI(R) was significantly enhanced (t-test, 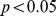) till 40% reduction (Figure 8C).

#### L2/3 SI cells enhance the attentional gain effect and the synchrony of L2/3 RS cell activity

**Figure 9:**
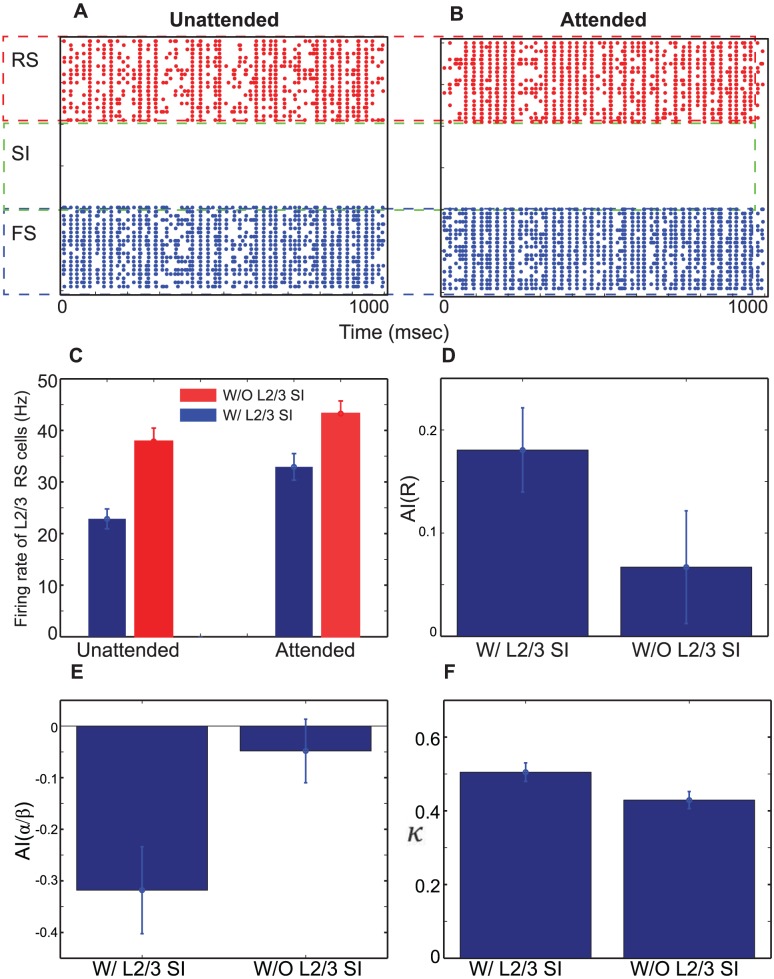
Functional roles of L2/3 SI cells. (A)–(B). Superficial activity without L2/3 SI cells. (C). The firing rate of L2/3 RS cells, with and without L2/3 SI cells. (D)–(E). Comparison of attentional indices, with and without L2/3 SI cells. For each index, bar graphs show mean values from 10 simulations, and errorbars are standard errors from 10 simulations. (F). Comparison of the synchrony in RS cell activity, with and without L2/3 SI cells.

To understand functional roles of L2/3 SI cells during the stimulus period, we removed L2/3 SI cells from our model. The removal of L2/3 SI cells produces two noticeable differences: First, the spiking activity of L2/3 RS cells is enhanced in both columns (Figure 9A, B and C), reducing AI(R) and AI(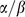), attentional effects on both firing rate of L2/3 RS cells (Figure 9D) and synchrony in the low frequency band (Figure 9E). Without L2/3 SI cells, AI(R) and AI(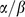) are not significantly different from 0 (t-test, 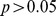). Second, the synchrony in firing of L2/3 RS cells decreases; we calculated the coherence to compare synchrony in firing of L2/3 RS cells of the attended column with and without L2/3 SI cells (see Methods). As can be seen in Figure 9F, the synchrony is significantly smaller (t-test, 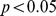) when L2/3 SI cells are removed. L2/3 SI cells can increase synchrony by preventing L2/3 RS cells from firing between cycles of gamma rhythms. At each cycle, L2/3 RS cells receive a strong excitation from L4 E and other L2/3 RS cells, and thus inhibition of L2/3 FS cells cannot stop L2/3 RS cells from responding to such an excitation. However, slow decaying inhibition of L2/3 SI cells can provide sustained inhibition capable of suppressing L2/3 RS cell activity induced by background inputs between cycles of gamma rhythmic ascending excitation from L4 E cells.

Simulation results without L2/3 SI cells suggest that L2/3 SI cells, associated with low-frequency power in STA of LFPs, help L2/3 RS cells to fire synchronously and suppress L2/3 RS cell activity in the unattended column.

#### FS cells also participate in the attentional gain modulation

Our model suggests the critical role of SI cells in attentional modulation of neural responses to bottom-up inputs, and one may ask whether FS cells are also involved in this process. In our model, FS cells do contribute to attentional modulation in laminar-specific ways. If L4 FS cells are hyperpolarized, activity of FS cells not provoked by pyramidal cell firing is reduced, and consequently, the effect of top-down attention on the gamma-frequency power is also reduced. If L5 FS cells are highly depolarized, L5 SI cell activity may be suppressed too strongly to allow significant ascending inhibition. We tested these by modulating tonic inputs (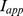) to FS cells in all layers. Specifically, we set 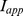 to 2.0 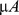, 1.0 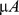 and −2.5 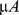 for L2/3, L4 and L5 FS cells, respectively; in our terminology negative 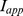 depolarized membrane potentials of cells (see Methods).

**Figure 10:**
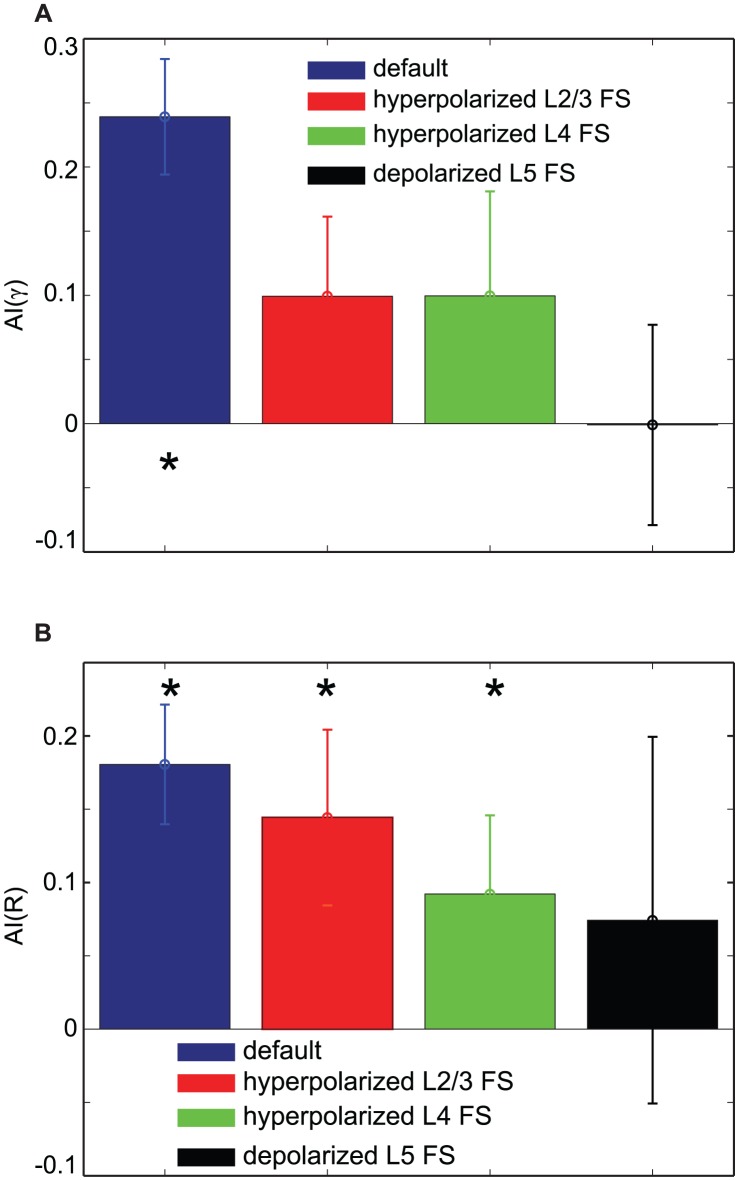
The effect of excitability of FS cells. Comparison of attentional indices. (A). The effect on the synchrony in the gamma frequency band. (B). The effect on the firing rate of L2/3 RS cells. * represents distributions significantly different from 0.

As can be seen Figure 10, hyperpolarization of L2/3 and L4 FS cells reduce attentional effects on synchrony in the gamma frequency band (Figure 10A) and firing rate of L2/3 RS cells (Figure 10B), and attentional effects are reduced when L5 cells are too depolarized.

#### Asynchronous top-down signals

**Figure 11:**
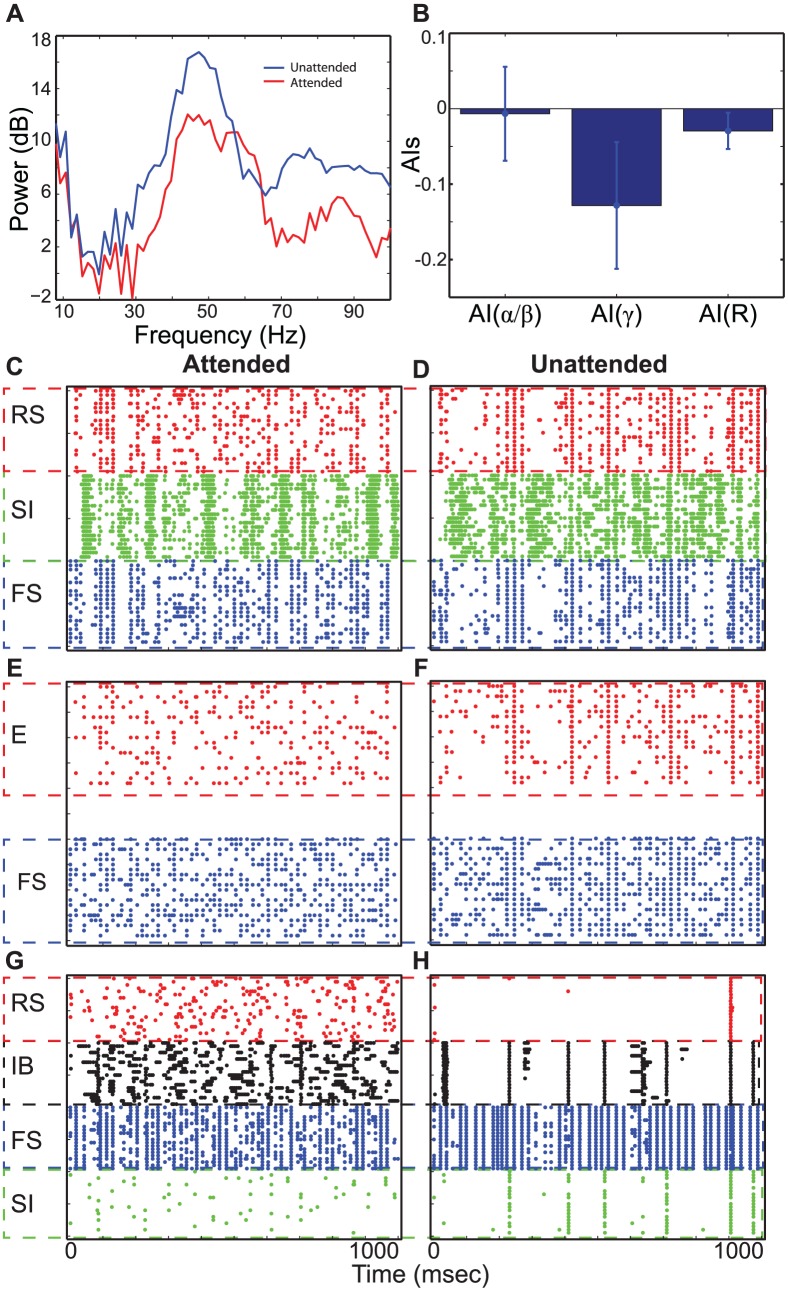
The effect of asynchronous top-down signals. (A). The power spectra of STA of LFPs with asynchronous top-down signals. (B). Attentional indices; all indices are not significantly different from 0. (C)–(H). Cell activity in both columns with asynchronous top-down signals.

We also tested whether asynchronous top-down signals could also enhance the neural response of the attended column. In this experiment, we replaced top-down beta rhythms with asynchronous 20 Hz Poisson trains of EPSCs. Results are strikingly different from those with synchronous top-down signals. As can be seen in Figure 11, asynchronous top-down signals reduce the synchrony in the gamma frequency band (Figure 11A and B). The reason is that asynchronous top-down signals provoke L5 pyramidal cells to fire independently from inhibition projected to them. Once L5 pyramidal cell activity becomes asynchronous, L5 SI cell activity is substantially reduced (Figure 11G). By contrast, in the unattended column, L5 IB cells fire sparsely but synchronously (Figure 11H). When IB cells fire, they are capable of inducing L5 SI cells to project strong ascending inhibition to L4 FS cells, generating stronger gamma rhythmic activity in the unattended column (Figure 11C, D, E and F). Hence, synchronous top-down signals are necessary to reproduce the Fries et al. [5] results.

## Discussion

Top-down signals in the beta frequency bands have been observed in various sensory systems (see Reference [23] and references therein), and potential links between top-down beta rhythmic activity and cognitive functions were reported in audition [21] and vision [18], [19], suggesting that the same mechanism may be associated with both visual and auditory signal processing. In addition, a similar interlaminar connectivity was found in multiple cortical areas [45]. Since the neural mechanism of the primary auditory cortex (A1) responsible for deep layer beta rhythms was well studied [24], we adopted the previously proposed deep layer model of A1 to study the potential mechanism by which top-down beta rhythms can modulate visual signal processing. In this section, we summarize our results and present implications and limitations of our model.

### Neural pathways responsible for attentional gain control

In this study, we introduced top-down signals to L5 pyramidal cells (see Methods), as suggested in Wang [23], but we also note that top-down signals can directly target L5 interneurons [35], [46]. Even if top-down signals prominently project to L5 interneurons, L5 SI cells are dominant over FS cells [29], and deep layer beta rhythms still emerge, producing the equivalent interlaminar interaction. Thus, we did not explore the effect of top-down beta rhythms projected to L5 interneurons.

Our simulation results suggested potential mechanisms for selective attention: L5 cells resonating to top-down beta rhythms can modulate superficial layer activity by generating ascending projection to upper layers. Specifically, we found exclusive roles for excitatory and inhibitory synaptic projections from L5 cells to upper layers. First, an inhibitory projection originating from L5 SI cells to L4 FS cells enhanced excitatory outputs of L4 in response to bottom-up inputs. Second, an excitatory projection from L5 IB and RS cells of the attended column generated stronger inhibition to L2/3 RS cells of the unattended column via intercolumnar connections to L2/3 interneurons of the unattended column. In our model these two types of ascending projections associated with top-down beta rhythms generated activity in superficial layers in accord with the pattern of neural response modulation induced by attention reported in Fries et al.[5], [6]: Compared with attention outside the RF, top-down beta rhythms associated with attention inside the RF can 1) enhance gamma rhythms in L2/3, 2) reduce beta rhythms in L2/3 and 3) enhance spiking activity of L2/3 RS cells. Since superficial layers project to higher cognitive areas, these modulatory effects of top-down signaling may account for the enhanced Granger causality influence from V1 to V4 reported in a recent *in vivo* study [20].

Importantly, SI cells in both L2/3 and L5 play major roles in our model of attentional gain control. First, L5 SI cells produced ascending inhibition. Second, L2/3 SI cells enhanced synchrony in L2/3 RS cells of the attended column and induced stronger inhibition in L2/3 of the unattended column (see Figure 9 and related text), indicating that L2/3 SI cells are critical for lateral inhibition. This active role of L2/3 SI cells is consistent with findings in Kapfer et al. [47] that adaptive interneurons, which include our SI cells, are critical to lateral inhibition. During the delay period, top-down beta rhythms suppressed RS cell activity induced by background inputs only (Figure 3B). We are not aware of any evidence for or against this prediction of our simulations.

In the model we have assumed that the top-down pathway have the necessary specificity. Indeed, long-distance connections are more target-specific than short-distance connections [48]. Though we do not address how specific connections are established among cortical areas (a huge question), our contribution is to show how these top-down pathways can dynamically regulate the gain of sensory signal processing.

### Distinctive roles of interneurons for cortical rhythms and signal processing

FS and SI cells of our model participate in generation of gamma and beta rhythms. What roles do these two types of interneurons play in signal processing? In a local circuit that generates gamma rhythms, inhibition from FS cells prevents RS cells from responding to synaptic inputs between cycles of gamma rhythms, a feature useful for filtering out distracting inputs [49]. Since L4 cells produce rhythmic activity in response to bottom-up inputs in our model and initiate gamma rhythms in L2/3, L2/3 FS cells can ensure L2/3 RS cells selectively respond to bottom-up inputs; this selectivity enhances signal to noise ratio of sensory signal processing significantly if the frequency of gamma rhythms in L2/3 match those from L4 [50]. This suggestion is consistent with the role of FS cells in generating gamma rhythms associated with sensory input [40], [51] and with the reduction of noise with gamma rhythms [52]. In contrast, our model suggests that SI cells, which have been thought to be critical in regulation of pyramidal cell activity [47], [53], may also be involved in modulatory control of FS cells and thus gamma rhythms. In particular, L2/3 SI cells provide additional inhibition to suppress erroneous L2/3 RS cell activity during the delay period, and L5 SI cells can enhance L4 outputs in the attended column.

### Surround inhibition and lateral inhibition

Surround suppression has been widely reported in visual systems, and the traditional view considers lateral inhibition as a key mechanism. However, this view has been recently challenged by studies suggesting a reduction of inhibition that is contradictory to the enhanced inhibition predicted with lateral inhibition in the cortex: Ozeki et al. [54] revealed that both inhibitory and excitatory inputs were reduced when surround suppression was in effect, leading them to conclude that surround suppression originated in the thalamus, rather than in the cortex. This issue is still under debate [55], [56]. Given the conflicting evidence, the two different mechanisms relying on different origins may coexist and cooperate with each other, as discussed in [57], [58].

Although several studies support crosstalk among cortical columns, this interaction may be distance-dependent: Adesnik & Scanziani [56] found that the lateral suppression effect *in vivo* in anesthetized animals faded in two columns. This could be a potential concern for our model since the unattended column may be located far away from the attended column in the brain. In such a situation, lateral inhibition may be weak and attentional modulation be significantly reduced, especially in the alpha/beta frequency bands (see Figure 9 and related text). However, it should be noted that monkeys in the Fries et al. studies [5], [6] had extensive training. During the training period, long-distance connections among columns could develop to improve performance. Alternatively, lateral inhibition, assumed in our model and other computational studies, may be conveyed indirectly to distant columns via the thalamus. L5 cells project strongly to the thalamus, which projects back to superficial layers globally; they also project to LGN, which has specific projections to the cortex [59]. Indeed, a few studies suggested that interneurons could be a main target of thalamocortical (TC) projections [60], [61]; see also Reference [62]. Thus, these two pathways may allow L5 pyramidal cells to create a global and local synaptic projection from L5 pyramidal cells to L2/3 interneurons through the thalamus.

Interestingly, many computational models have proposed active roles of lateral inhibition for attentional gain control [10], [16], [17], [63] (see below), different from involuntary surround suppression; top-down signals, selectively projected to a population of cells, can assist the selected population to be a winner in the competition with other populations via lateral inhibition. For instance, in the model proposed by Ardid et al. [64], excitatory to interneuron connections, uniformly implemented throughout the cortices, gave rise to biased competition; such structure is consistent with lateral connections in our model. Our model is in line with those models, in that top-down signals can bias competition. However, we add the novel proposal that interlaminar interaction, regulated by top-down beta rhythms, support selective attention. Lateral connections from L5 pyramidal cells to L2/3 interneurons in our model were adopted from the hierarchical architecture proposed by Felleman & Van Essen [34]. Indeed, most L5 pyramidal cells were also found to project into L2/3 [65], [66]; L5B projects to L2 and L3A with widely branched axons [37].

### Top-down signals in the gamma frequency band

Although our study focused on functional roles of top-down beta rhythms and their underlying mechanisms, top-down gamma rhythms have also been reported in a few studies [24], [25]. Since FS cells are believed to play a critical role in gamma rhythms, top-down gamma rhythms may be associated with modulation of FS cell activity. This assumption is supported by two physiological studies. First, Mitchell et al. [4] revealed that top-down attention enhanced FS interneuron activity most strongly. Second, Roopun et al. [24] found that superficial and deep layers received top-down gamma and beta rhythms, respectively. Together, we hypothesize that top-down gamma rhythms mainly target superficial FS cells.

What are the functional roles of top-down gamma rhythms? Roopun et al. [24] noted that L2/3 gamma rhythms of S2 entrained L2/3 gamma rhythms of A1. This entrainment makes A1 mirror superficial activity of S2 rather than auditory inputs from L4 of A1, and thus A1 cannot project its own auditory signals to S2 when prominent top-down gamma rhythms exist. This means that cortico-cortical projections from A1 to S2 may be disabled by top-down gamma rhythms. Thus, we further hypothesize that top-down gamma rhythms may be responsible for routing/gating feedforward processing related to bottom-up inputs, depending on attentional state. This hypothesis is explored in a forthcoming paper addressing interaction between top-down gamma and beta rhythms.

### Alpha rhythms

We note that alpha rhythms have also been reported to be associated with suppression of neural responses induced by irrelevant stimuli when attention was demanded [67]–[69]. Spaak et al. [70] found a prominent alpha rhythmic activity in deep layers, and Buffalo et al. [28] reported a reduction of SFC in the alpha frequency band during the attention inside the RF trials. It is widely accepted that alpha rhythms are generated at least partly in the thalamus; Saalmann et al. [71] found that the pulvinar indeed regulated the alpha rhythmic activity in V1 and V4 and the coherence between them in the alpha frequency band, suggesting that attention-associated alpha rhythms may be induced by the thalamus.

Since our model does not include the thalamus, we cannot explicitly test whether thalamic projections in the alpha frequency band can account for findings of Buffalo et al. [28], but our model provides a perspective on the possibility: In our model, SFC in the alpha frequency band (8–15 Hz) was significantly (t-test, 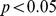) smaller in deep layers of the attended column than in deep layers of the unattended column, whereas SFC in the beta frequency band (15–25 Hz) was significantly larger in the deep layer of the attended column (Figure S1). In the attended column, top-down beta rhythms entrained L5 cells, reducing the alpha rhythmic activity, which appeared in the deep layers of the unattended column as well as the control column. In other words, top-down beta rhythms to L5 cells competed with alpha rhythms and reduced the alpha rhythmic activity in deep layers, suggesting that top-down beta rhythms can reduce the impact of thalamic alpha rhythms via competition in the attended column.

This raises a critical question: Why are both alpha and beta rhythms necessary for attentional modulation? We suggest that these two rhythms may play complimentary roles: Olsen et al. [72] suggested that L6, which receives thalamic inputs [73], suppresses neural activity. If the thalamus broadly projects alpha rhythms into L6 of the cortex, the cortical neural activity may be suppressed globally. For example, the pulvinar projects to superficial layers of V1 and granular layers of V2 [74], and L6 pyramidal cells can receive focused inputs from superficial and granular layers [73], suggesting that the pulvinar may be capable of inducing global inhibition via L6 cells. By contrast, our model proposes that L5 cells can enhance neural response within a specific column via ascending inhibition. Together, we propose a scenario that alpha rhythms may suppress neural responses globally, whereas beta rhythms can release a particular column/receptive field from global suppression.

### Implications for functional roles of ACh

Acetylcholine (ACh) is a neuromodulator associated with attention (see Reference [26] for a review). Hasselmo [75] and Kimura [76] found that ACh suppresses neural transmission originating from the cortex, allowing cortical cells to be more sensitive to transmission from thalamus. However, it is still unclear how the highly diffuse projections of ACh-releasing terminals throughout cortex can support selective attention. Our model proposes a mechanism by which ACh may contribute to selective attention: Xiang et al. [29] found that ACh can depolarize L5 LTS cells via nicotinic receptors and hyperpolarize L5 FS cells via muscarinic receptors. Since FS cells inhibit LTS cells, both of these effects make the LTS cells with vertical axons, a subset of interneurons known to provide slow inhibition [77], resonate more easily to top-down beta rhythms, enhancing biased competition in superficial layers. Thus, the diffusive projection of ACh changes the network to facilitate top-down signaling in providing these selective signals.

When ascending inhibition of L5 SI cells and L2/3 SI cells were removed from the model, attentional gain control disappeared; this indicates a critical role of nicotinic receptor regulation in selective attention because SI cells are depolarized by ACh via nicotinic receptors [29], [78]–[80]. Indeed, a link between nicotinic receptors and attention was supported by a few studies [81]–[83]; see Reference [84] for a review. Changes in nicotinic receptor regulation may thus be important for understanding cognitive changes in some diseases associated with attention deficit [85]–[90]. Importantly, Levin [91] found that nicotine alleviates some symptoms of Attention-Deficit/Hyperactivity Disorder (ADHD), and the procognitive effects of cigarette smoking found in patients with various psychiatric disorders are well documented (see Reference [92] for a review). Although further studies should be conducted to investigate how nicotinic receptor regulation is linked to such psychiatric disorders, our model raises the possibility that impaired nicotinic receptor function can cause imaginary sensation: the removal of L2/3 SI cells from our model produced gamma rhythmic activity in L2/3, independent of bottom-up inputs. If these gamma rhythms are misinterpreted by higher cognitive areas as resulting from stimulus presentation, they may help to account for hallucination, a symptom of some psychiatric disorders. Interestingly, Manganelli et al. [93] reported evidence suggesting that visual hallucination is caused by impaired cholinergic systems.

Herrero [94] found evidence that muscarinic receptor blockers can reduce attentional focus. This phenomenon may be attributable to cholinergic switching in L5 [29]: These authors found that muscarinic receptor blockade depolarized L5 FS cells. When we simulated this effect, LFP modulation induced by top-down signals was reduced (see Results), consistent with Herrero et al. [94]. However, more complex, equivocal reports of muscarinic receptor-mediated effects on fast-spiking interneurons need to be considered as a caveat to this [80], [95]–[97].

### Limits of our model

Most spontaneous layer-specific data was collected from anesthetized animals, and its link to *in vivo* activity from awake animal engaging in cognitive tasks, is currently unclear. Since our model aimed to understand mechanisms underlying top-down attentional effects, we assumed our model was cholinergically modulated; we depolarized L5 SI cells and hyperpolarized L5 FS cells in deep layers [29] during all simulations including the control experiments, suggesting that spontaneous activity collected from anesthetized animals cannot be used to tune our model network behaviors.

Instead of capturing spontaneous *in vivo* activity of L4 and L5 cells, we considered L4 and L5 as simple recipient layers for bottom-up and top-down inputs, respectively, and did not introduce any external Poisson spike trains of EPSCs (external background inputs). Due to this simplification, during the delay period, L4 E cells did not have sufficient drive to overcome inhibition from L2/3 and L4 FS cells (see Figure 1) and were quiescent. We evaluated the impact of the lack of drive to L4 cells by introducing external background inputs to L4 E cells and found no significant changes: L4 E cell activity was increased with background inputs simulated by 10 Hz Poisson EPSCs with various amplitudes (Figure S2A), but attentional indices still showed significant attentional modulation during the stimulus period (Figure S2B, C and D).

We also note that L5 pyramidal cells fired quite synchronously in the alpha frequency band even with external background inputs, during the control experiments, since they received inhibition from L5 SI cells. It is important to note that a few studies indeed found alpha rhythms in deep layers [28], [70], [98] while animals were awake, consistent with synchronous activity in L5 of our model. In addition, Berger et al. [99] suggested that firing of a few L5 pyramidal cells can induce feedback inhibition, and L5 Martinotti cells, a class of SI cells, were reported to provide feedback inhibition between L5 pyramidal cells [53], suggesting that synchronous activity of pyramidal cells can approximate *in vivo* spontaneous activity in deep layers.

Our model ignored two components. First, we considered only two columns, although a lot of “inactive” columns, driven weakly by bottom-up inputs, interact with the attended and unattended columns. Second, the thalamus was missing in our model, making us unable to evaluate the effect of thalamocortical circuits on attentional modulation. Since the thalamus is known to generate alpha rhythms [71], [100], the thalamocortical interaction should be taken into the account to fully understand mechanisms underlying the prominent alpha rhythms and reduction of alpha rhythms reported in Buffalo et al. [28]. In spite of these simplifications, our model was capable of reproducing attentional modulation reported in Fries et al. [5], indicating the importance of beta-rhythmic cortico-cortical communication on attentional gain control.

### Comparison with other computational models

The importance of selective attention in cognitive abilities has attracted multiple computational model studies. Most of these studies concern competition between multiple stimuli in the same field. In particular, Reynolds et al. [10] presented both neurophysiological data supporting biased competition and a firing rate model capable of reproducing their findings. Since then, more biophysically detailed models have been proposed to study neural mechanisms underlying biased competition. These include Deco & Rolls [63], who built a model of V2 and V4; their simulation results provided a potential mechanism underlying the competition between bottom-up and top-down regulations. Similarly, resonance between MT and PFC were shown to be able to generate multiplicative gain modulation [64]. Buia & Tiesinga [16] proposed a theory that top-down signals are delivered to interneurons, instead of excitatory pyramidal cells. The effects of top-down attention on cortical columns with lamina structure were investigated in Wagatsuma et al. [17]. Tiesinga & Sejnowski [101] showed that synchrony in interneurons can regulate signal flow; also, gamma rhythms can enhance the selection of relevant stimuli [49].

By contrast, our model, which focuses on reproducing neurophysiological data reported in Fries et al. [5], involves comparing an ‘attention-inside’ condition to an ‘attention-outside’ condition. Our model is most closely related to the model proposed by Buia & Tiesinga [16], in that interneurons, activated by top-down signals, play crucial roles in attentional gain control. However, in addition to discussing a different paradigm (competing inputs), their model has no laminar structure, which is critical for the effects of our model. Our model suggests that the laminar structure can ensure that L2/3 RS cells, which project to higher cortical areas, respond to bottom-up inputs more strongly and reliably when attention is directed to the column. Wagatsuma et al. [17] used a model with laminar structure similar to ours, but without biophysical details we believe are critical to the phenomena we describe.

In summary, focusing on the detailed physiology of the network receiving top-down input enabled us to suggest mechanisms for the action of those inputs in facilitating attentional gain control. The model further sheds light on how diffuse cholinergic modulation can support selective attention.

## Methods

### Numerical simulations

As can be seen in Figure 1, our model consists of nine cell populations with randomized connections among them. We ran 10 independent simulations for each condition to reduce bias from random connections and noise. To do so, we reduced the complexity of the model by limiting each population to have 20 cells, as in Kramer et al. [32]. All integrations were numerically calculated by the fourth-order Runge-Kutta methods with 0.01 msec-time step. Noisy tonic input 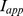, injected to each neuron, generated heterogeneity in cells of the same type. This noisy current 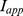 was produced using the Box-Muller algorithm [102] (see Table 1). Simulation codes were written in C.

**Table 1:** The maximal conductances intrinsic currents and external inputs.

	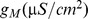	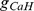	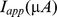	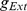
L2/3 RS	0.5	0	0(0.5)	0.2
L2/3 FS	0	0	0(0.5)	0.02
L2/3 SI	8	0	−1(0.5)	
L4 E	0.3	0	−1(0)	1.0
L4 FS	0	0	2(0.5)	0.03
L5 IB (axon)	2	0	1(0.1)	
L5 IB (soma)	0	0	1(0.1)	
L5 IB (dendrite)	4	4	2(0.3)	3.0
L5 RS (axon)	2	0	1(0.1)	
L5 RS (soma)	0	0	1(0.1)	
L5 RS (dendrite)	4	1.6	2(0.3)	3.0
L5 FS	0	0	0(0.5)	
L5 SI	4	0	−1(0.8)	

For each compartment, noisy tonic drive (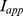) was introduced. We display mean values; the numbers inside parentheses are standard deviations.

### Neuron models

All L2/3 and L4 cells were modeled with single compartments. Pyramidal and SI cells contained a transient sodium (NaF) current, a delayed rectifier current (KDR), a leak current and a muscarinic current (M). The model FS cells did not contain a M-current, which provides frequency-adaptation [97], [103]: they contained NaF, KDR, leak currents as in Wang & Buzsaki [104]. Deep layer SI cells were modeled the same as L2/3 SI cells except the maximal conductance of M-current, which was reduced by 50%. However, L5 pyramidal cells (IB and RS) were modeled with three compartments (axon, soma and apical dendrite) to reflect the fact that L5 pyramidal cells with apical dendrites can receive top-down signals [37]. These three compartments were connected via electrical coupling as in Kramer et al. [32]. Each compartment contained NaF, KDR, and leak currents. M-current was included in both dendrite and axon, and high-threshold calcium (CaH) current was added to the dendrite. IB cells contain higher maximal conductance of CaH current than RS cells [38].

Dynamics of neurons in our model were calculated by voltage-gated conductance equations;
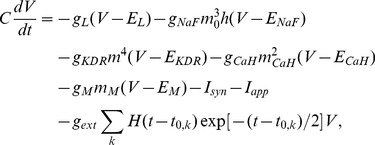
(1)where 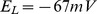, 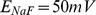, 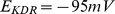, 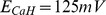 and 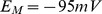; where the last term represents Poisson trains of EPSCs: 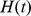 is the Heavyside step function and 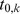 are the arrival times of trains of EPSCs (see below). Membrane potentials were regulated mainly by two different sources; intrinsic ion currents and synaptic currents. Also we introduced two types of external inputs 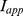 and trains of EPSCs with maximal conductance of 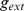. For all compartments (cells) except L2/3 RS cells, we used 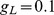, 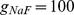 and 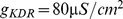; we lowered 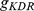 by 50% for L2/3 RS cells. Table 1 shows the maximal conductances of other currents and the mean value (standard deviations) of 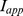 for each type of cell.

### Intrinsic ion currents

The gating variables 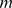 and 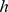 regulating ion currents follow Hodgkin-Huxley-type equations.
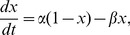
(2)where 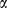 and 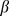 are forward and backward rate functions. With the relationships between forward and backward rate functions and steady state variables;
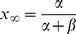
(3)

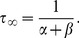
(4)
Equation 2 can be described with steady-state variable;
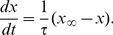
(5)We adopt steady-state variables for NaF, KDR and CaH currents from Kramer et al. [50]. Excitatory and inhibitory cells are different in dynamics of NaF and KDR currents. For excitatory cells and compartments, we set steady-state variables as;
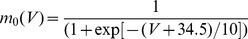
(6)

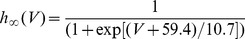
(7)

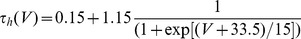
(8)

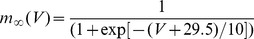
(9)

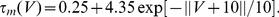
(10)For inhibitory cells, we used slightly different steady-state variables;
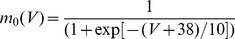
(11)

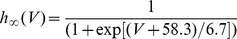
(12)

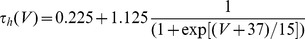
(13)

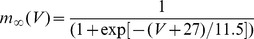
(14)

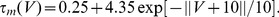
(15)For M-current, we adopted the forward and backward rate functions from McCarthy et al. [105];
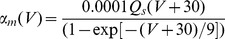
(16)

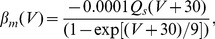
(17)where 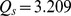. Similarly, for CaH current, we set the forward and backward rate functions following Kramer [32];
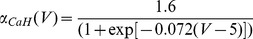
(18)

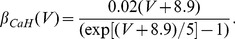
(19)


### Synaptic connections

In each layer, all pyramidal cells and inhibitory interneurons were reciprocally connected, and recurrent excitatory and inhibitory connections were also implemented; thus cells received both excitation and inhibition from other cells belong to the same layer. 25% L2/3 FS and SI cells had NMDA receptors and thus they received excitatory inputs from L2/3 RS cells via both NMDA synapses and AMPA synapses. We did not consider NMDA-mediated excitatory connections among L4 E cells in this study since most outputs of L4 neurons in visual cortex of guinea pigs show fast kinetics, decaying within 2 msec [106]. The rise and decay time of synapses are shown in Table 2.

**Table 2:** Rise time and decay time of synapses used in our model.

	rise (msec)	decay (msec)
excitatory synapses	0.25	1
inhibitory synapses from FS cells	0.5	8
NMDA synapses	5.0	100
inhibition from SI cells	0.5	20
excitation from L5 pyramids to L2/3 SI	2.5	50

Non-fast spiking interneurons elicited slowly decaying inhibition [77], [113], and inhibition of L5 Martinotti cells has slower kinetics than that of L5 FS cells [53]. Thus, we set SI to produce slowly decaying inhibition. Specifically, we adopted decaying time from models of low threshold spiking interneurons in earlier computational works [32], [38].

For interlaminar connections, we used a subset reported in neurophysiological studies [37], [107]. Instead of implementing all synaptic connections reported, we focused on identifying synaptic pathways responsible for attentional gain control of sensory signal processing. Figure 1A shows the structure of the cortical column. We connected layers with excitatory and inhibitory synapses;

- Connections between L2/3 and L4: L2/3 RS cells received excitatory and inhibitory inputs from L4 E and FS cells, respectively, whereas L2/3 FS cells received excitatory inputs only. Although a prominent excitatory projection from L2/3 to L4 was found in the primary auditory cortex, it was rarely found in other sensory cortices [107]. Thus, we did not implement excitatory projections back to L4 from L2/3. In contrast, L4 E cells received inhibitory projections from L2/3 FS cells.
- Connections between L2/3 and L5: L2/3 RS cells sent descending excitatory projections to L5, and L5 pyramids projected to L2/3 SI and FS cells. As in Kramer et al. [32], synapses connecting L5 pyramids to L2/3 SI cells decayed much more slowly than those connecting L5 pyramids to L2/3 FS cells: the rise and decay times of synapses connecting L5 pyramids to L2/3 SI were 2.5 and 50 msec, respectively, whereas synapses conveying postsynaptic inputs to L2/3 FS cells had 0.25 msec-rise time and 1.0 msec-decay time.
- Connections between L4 and L5: L4 E cells projected excitatory synaptic inputs to L5 pyramids (RS and IB) and FS cells. Since L5 Martinotti cells have vertical axons [29], [30], [37], [108], we connected SI cells to L4. L5 SI cells produced ascending inhibitory inputs to L4 FS cells since one of main targets of L5 Martinotti cells is L4 [108]. L5 SI cells inhibited L4 FS cells only in the model.

Two columns were connected with each via ascending excitation (Figure 1B). The intercolumnar connections to L2/3 SI cells were 50% stronger than those within the column.

Table 3 shows details of the connectivity of our model. We considered each synapse as a gating variable that controls the conductance of synaptic currents on a post-synaptic neuron. Each synaptic variable 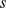 was simulated by the same rule:
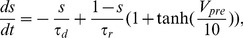
(20)where 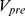 the membrane potential of pre-synaptic cell. 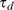 and 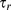 determine how fast synapses can close and open, respectively, and thus they were referred to as the decay time and rise time, as in Kramer et al. [32]. For a post-synaptic cell, synaptic input is the sum of excitatory and inhibitory synaptic currents:
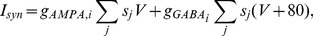
(21)where 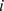 the is index for type of pre-synaptic cells (out of 9 types of cells in our model) and 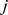 is the index for neurons in the population of the same type. The maximal conductance, rise time and decay time of individual synapse depends on both pre-synaptic and post-synaptic cell types, and chosen values are given in Table 2. In our model NMDA synapses were simulated with slow rise and decaying time constants (Table 2).

**Table 3:** Connectivity map.

# presynaptic cells		L2/3			L4		L5			
( 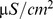 )		RS	FS	SI	E	FS	IB	RS	FS	SI
L2/3	RS	5	10	10	0	0	20	20	0	0
		(0.22)	(0.3)	(0.03)			(0.212)	(0.212)		
	FS	5	8	5	5	0	0	0	0	0
		(0.4)	(0.6)	(0.1)						
	SI	5	5	0	0	0	0	0	0	0
		(0.1)	(0.2)							
L4	E	5	0	0	10	10	10	10	20	0
		(0.2)			(0.4)	(0.2)	(0.212)	(0.212)	(0.3)	
	FS	5	0	0	10	10	0	0	0	0
		(0.02)			(1.0)	(0.3)				
L5	IB	0	2	2	0	0	10	10	10	10
			(0.2)	(0.2)			(0.02)	(0.02)	(0.12)	(0.12)
	RS	0	2	2	0	0	10	10	10	10
			(0.2)	(0.2)			(0.02)	(0.02)	(0.05)	(0.15)
	FS	0	0	0	0	0	20	20	20	10
							(0.1)	(0.1)	(0.5)	(0.3)
	SI	0	0	0	0	10	20	10	10	20
						(0.4)	(0.3)	(0,3)	(0.6)	(0.4)

In our model, each post-synaptic cell received synaptic inputs from various types of cells. The rows show the the type of post-synaptic cells, whereas columns represents pre-synaptic cells. We list how many pre-synaptic cells of a particular type were connected to a post-synaptic cell. For instance, L2/3 FS cells received excitation from 2 L5 IB cells. The numbers inside parentheses are maximal conductance of corresponding synapses. Additionally, L2/3 FS and LTS cells received excitation from 10 L2/3 RS cells via NMDA synapses; the maximal conductances are 0.04 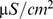 and 0.03 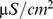, respectively. Since Roopun et al. [24] suggested that only L5 SI cells produced inhibition oscillating in the beta frequency band and that all L5 cells received beta rhythmic inhibition, all L5 cells of our model received inhibition from L5 SI cells. Also, we made inhibitory connections among L2/3 SI cells sparse and weak, since they are known to be rare [103].

### External inputs

To understand the modulatory effect of top-down signals on bottom-up sensory signal processing, we introduced top-down and bottom-up signals to our model. In this study, we did not explicitly implement higher cognitive areas or the thalamus, the sources of top-down and bottom-up signals. Instead, we assumed that top-down signals were synchronous synaptic inputs and bottom-up inputs were asynchronous ones. Specifically, both top-down signals and bottom-up inputs were simulated as trains of excitatory post synaptic currents (EPSCs), which caused instantaneous increase of AMPA current up to a maximal conductance and exponential decay with a 2 msec time-constant: Top-down signals were synchronized and oscillating at 20 Hz, and thus all L5 pyramidal cells of the attended column received the same EPSC trains; we did not introduce top-down signals to deep layer interneurons. In contrast, L4 E and FS cells of both columns received asynchronous 100 Hz EPSC trains generated from Poisson processes. We also introduced additional 50 Hz Poisson trains of EPSCs to L2/3 RS cells to simulate inputs from other parts of cortex [44], [109] and non-selective thalamic inputs such as matrix projections [110]; these inputs are referred to as background inputs in Results. Table 1 shows the maximal conductance values of external inputs.

### Simulation protocol

We ran the network for 1 sec each during the delay and stimulus periods. To simulate the delay period (after the cue and before the presentation of a stimulus), we introduced top-down signals into the attended column with no bottom-up input. In contrast, during the stimulus period, bottom-up inputs were introduced to both columns, with the attended column continuing to receive top-down signals. As control experiments for both periods, we considered simulations without top-down inputs; only background inputs were introduced to our model in a control experiment for the delay period. In order to uncover functional roles of SI cells and lateral inhibition, we also ran simulations removing such components from our model.

For each set of simulations, we created 10 realizations of networks with randomly chosen connections among cells (Table 3) and ran each of them with independently generated EPSC trains to minimize any bias from random connections and stochastic EPSC trains.

### Analysis of neural responses

The firing rate of cells is the average number of action potentials produced per second by 20 cells of each type, and we reported mean and standard errors of those values from 10 simulations. LFPs were simulated by summing up synaptic currents projecting onto L2/3 RS cells from all neurons in our model [111], and spike-triggered average (STA) of LFPs were calculated. In order to calculate STA of LFPs, we collected segments of LFPs surrounding each spikes of L2/3 RS cells from 10 independent simulations; thus, the simulated STA is averaged over spikes from 200 L2/3 RS cells. Specifically, we took 600 msec-long LFP segments; 300 msec before and 300 msec after each spike. All spectral analysis is done with Chronux, a matlab toolbox [112].

For each simulation run, we also calculated the spike-field coherence (SFC), a ratio of the power spectrum of STA of LFPs over the average of spike-triggered spectral spectra: This measure allows us to evaluate the synchrony of cell activity independently from the firing rate and spectral power of raw LFPs [5]. We reported mean values and standard errors of attentional indices from 10 simulations to show the modulatory effect of top-down beta rhythms [31]; three attentional indices are given as:
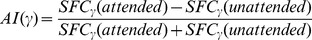


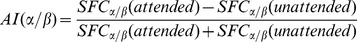


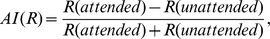
where 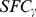 is the spike-field coherence in the high frequency band, 25–70 Hz, calculated from each simulation run; 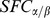 is the spike-field coherence in the low frequency band, 8–25 Hz; 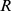 is the average firing rate of L2/3 RS cells from each simulation run.

We also measured synchrony of L2/3 RS cell activity with Equation (22) [104]

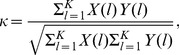
(22)where 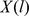 and 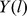 are the spike trains of two RS cells with 1-msec resolution.

## Supporting Information

Figure S1 **Attentional indices in the alpha and beta frequency band.** Attentional effects were measured from L5 pyramidal cells and deep layer LFPs in the alpha (8–15 Hz) and beta (15–25 Hz) frequency band, respectively.
(EPS)

Figure S2
**The impact of background inputs to L4 E cells on attentional modulation (A).** The mean value and standard errors of L4 E cells, induced by 20 Hz Poisson EPSCs. Attentional indices with various amplitudes of EPSCs. AI(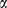/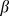), AI(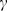), AI(R) are displayed in (B),(C) and (D), respectively. * represents distributions significantly different from 0.(EPS)
